# Light Induces Carotenoid Biosynthesis-Related Gene Expression, Accumulation of Pigment Content, and Expression of the Small Heat Shock Protein in Apple Fruit

**DOI:** 10.3390/ijms23116153

**Published:** 2022-05-31

**Authors:** Van Giap Do, Youngsuk Lee, Hunjoong Kweon, Seonae Kim

**Affiliations:** 1 Apple Research Institute, National Institute of Horticultural and Herbal Science, Rural Development Administration, Gunwi 39000, Korea; kongfo@korea.kr; 2 Postharvest Technology Division, National Institute of Horticultural and Herbal Science, Rural Development Administration, Wanju 55365, Korea; kweonhj@korea.kr

**Keywords:** apple fruit, coloration, pigment content, light, sunburn, small heat shock proteins

## Abstract

The coloration of the apple fruit (*Malus × domestica* Borkh.) depends on pigment content. Light stimulus activates a broad range of photosynthesis-related genes, including carotenoids. The effect of light on two red commercial apple cultivars, ‘Summer Prince’ and ‘Arisoo’ at the juvenile stage were examined. Apple fruits were either bagged to reduce light irradiation or were exposed to direct, enhanced sunlight (reflected). The pigment content and the expression of carotenoid metabolism genes in the peel and flesh of apple fruits were significantly different between the shaded and the reflected parts. These parameters were also different in the two cultivars, highlighting the contribution of the genetic background. Further, a combination of light and transient overexpression of carotenogenic genes increased fruit coloration and pigment content in the variety ‘RubyS’. Western blot analysis showed the expression of small heat shock proteins (smHSP) in lysates extracted from the reflected part of the fruits but not in the bagged fruits, indicating the activation of smHSP in response to heat generated by the reflected light. Therefore, the synergy between the genes and the environment dictates the color of apple fruits.

## 1. Introduction

The coloration level of apple fruits attracts consumers’ attention owing to their antioxidant capacity and health benefits. The content of pigments, especially carotenoids, plays a vital role in determining the color of the fruit. Carotenoids are known to provide many health benefits such as the prevention of cancer and cardiovascular diseases [1,2,3,4]. Given its biological activity, the use of carotenoids in foods and nutritional supplements has also been studied and well-reviewed [5,6,7]. The carotenoid biosynthesis pathway and its metabolic enzymes are well characterized in higher plants [8,9,10]. The first step is the geranylgeranyl diphosphate (GGPP) biosynthesis from pyruvate and glyceraldehyde-3-phosphate (GAP) via the non-mevalonate pathway. Next, the GGPP is catalyzed to phytoene by the enzyme phytoene synthase (PSY). Before the production of α-carotene and β-carotene, lycopene is produced from phytoene by phytoene desaturase (PDS), ξ-carotene desaturase (ZDS), and carotenoid isomerase (CRTISO) [11].

Carotenoid accumulation results from the regulation of biosynthesis, degradation, and stable storage, controlled by gene transcription and translation in fruits and other organs [12]. The structural genes encoding metabolic enzymes in the carotenoid pathway have been characterized [10]. Moreover, identifying the carotenoid biosynthetic genes in plants has boosted the nutritional value of different crop species through metabolic engineering approaches. A successful example of improving crop nutritional value is ‘Golden Rice,’ with a high content of vitamin A that was heterologously produced by incorporating two *PSY* genes [13]. PSY encoding is the first and plays an important role in the regulation of the carotenoid biosynthesis pathway. In the fruits of tomato and pepper, a change in color from green to red was concurrent with the increased production of lycopene and positively correlated with the enhanced transcription of upstream genes in the carotenoid biosynthesis pathway such as *PSY* and *PDS* [14,15,16]. Overexpression of *PSY* led to an increase in levels of total carotenoids in tomato fruits [17], maize [18], rice [19], pepper [20], tobacco [21], and apple [22,23]. Additionally, the heterologous overexpression of *LcPDS*, *LcZDS*, and *LbZDS* enhanced carotenoid accumulation in transgenic tobacco [21,24]. Moreover, carotenogenic gene expression can be influenced directly or indirectly by other proteins [25,26] and transcription factors [27].

Light is an important environmental factor involved in regulating the carotenoid biosynthesis hence influencing fruit coloration [28]. Both light quantity (intensity and duration of light exposure), and quality (spectral wavelength) affecting the accumulation of carotenoid contents have been reviewed [29]. Modulation of carotenoid biosynthesis and its accumulation in response to light has been recently reviewed [30]. Carotenoids play essential functions in photosynthesis for photosystem assembly, light-harvesting, and photoprotection [31]. Light influences fruit pigment accumulation by regulating the expression of carotenoid biosynthesis-related genes through light perception, transcription factors (PIFs and HY5), and light signaling repressors (COP1, CUL4-DDB1 E3 ligase) [30,32]. In *A. thaliana*, PIF1 directly binding to the promoter of the *PSY* gene downregulates carotenoid accumulation [33]. In tomatoes, PIF1-regulated carotenoid biosynthesis during fruit ripening represses carotenoid accumulation conditions by binding to the promoter of *PSY1* [32]. Another member of the PIF family, PIF3, is known to decrease carotenoid accumulation by negatively regulating the expression of carotenoid structural genes also evaluated in peach [34]. Moreover, light is also involved in the regulation of the transcript expression of anthocyanin biosynthesis-related genes [35]. Therefore, light could regulate pigment content through a combination of two biosynthesis pathways.

Fruit coloration is positively correlated with light levels. However, high solar radiation and hot air temperatures produced heat-damaged apple fruits with sunburn symptoms. Like other plants, apples accumulate heat shock proteins (HSPs) in response to heat stress. HSPs are molecular chaperones involved in many critical metabolic processes and play essential roles in responding to high temperatures. Under high temperatures, HSPs function to protect proteins from denaturation and aggregation, stabilizing their structure and maintaining quality [36,37,38]. Several classes of HSPs are known to exist in plant cells, and their expression is induced by heat. They can be divided into two classes based on their molecular weights. The first class contains high molecular weights such as HSP100 and HSP70, and the second class has low molecular weights between 15–30 kDa like the small heat-shock proteins (smHSPs). The first class of HSPs is highly conserved and constitutively expressed, while smHSPs are less conserved and rarely expressed in the absence of heat stress. Moreover, smHSPs are abundant proteins expressed during heat stress [39].

The regulation of carotenoid biosynthesis in apples has been investigated under the influence of either genetic or environmental factors. However, the impact of these two additive factors on color and pigment accumulation has not been studied and fully understood. Herein, we investigated the influence of both genetic (gene expression) and environmental (light) factors and determined the more influential factor. We found that environmental and genetic factors together stimulate the coloration of apple fruit and increase the accumulation of pigment content. Nevertheless, the role of external factors is more pronounced than internal factors. These findings will help better understand the molecular mechanism of carotenoid accumulation in apples under the effect of environmental and genetic factors.

## 2. Results and Discussion

### 2.1. Different Coloration Patterns of Apple Fruit under Light Treatment

**Figure 1:**
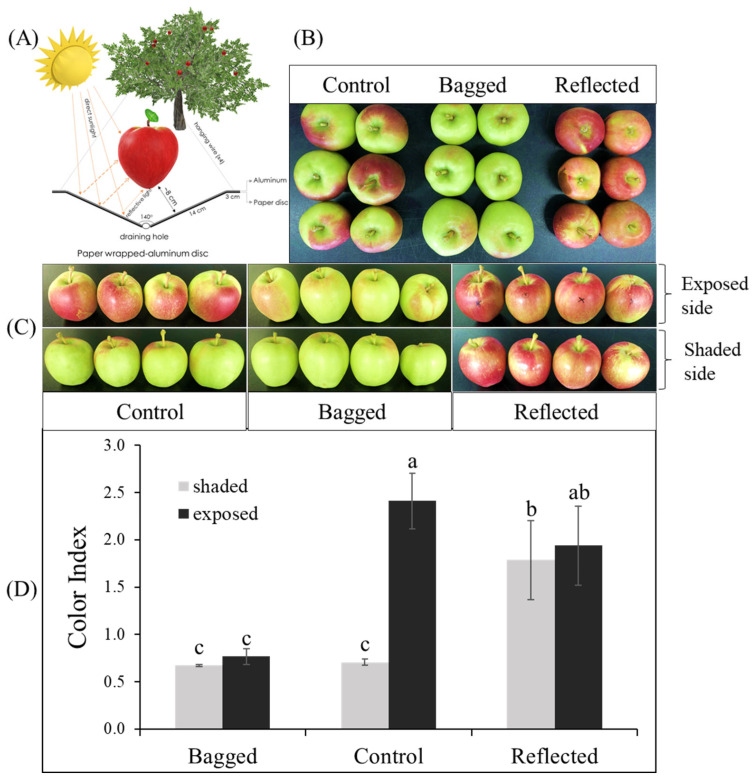
For the reflected sunlight treatment, the fruit was set up with a paper wrapped-aluminum disc to increase light intensity on the fruit of apple ‘Arisoo’ (**A**). The fruits showed a different coloration pattern between the three groups: reflected sunlight, bagged, and control (**B**), and between the exposed and shaded sides in the same fruit (**C**). The red-coloration level was positively correlated with color index (**D**). Different letters above the bars indicate a significant difference (*p* < 0.05). Data are presented as mean ± SD (*n* = 20) of three biological replicates.

**Figure 2:**
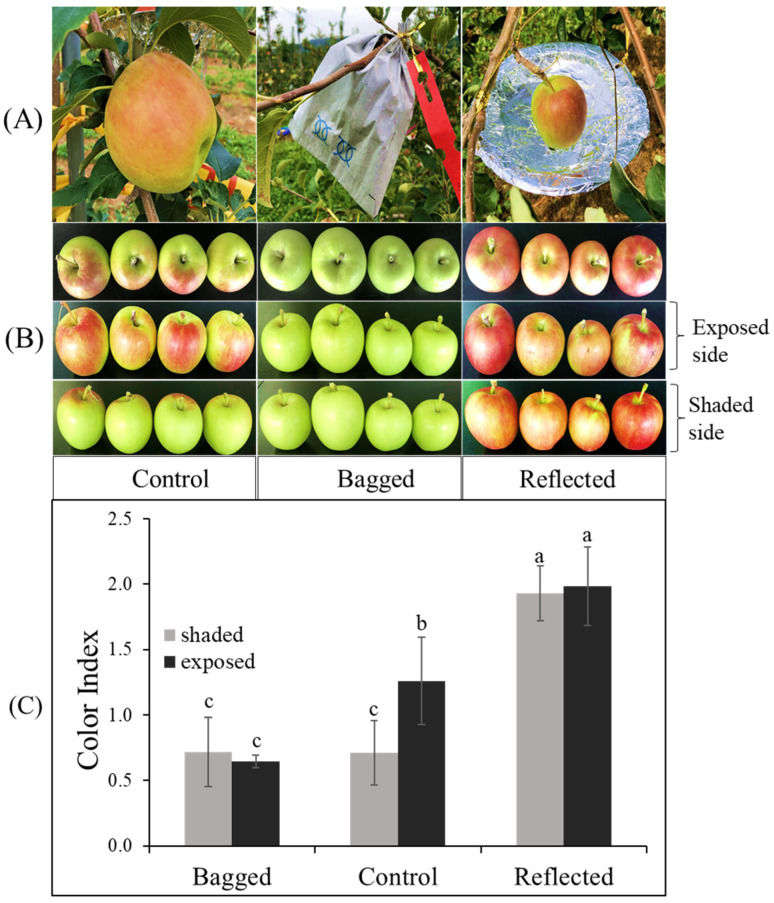
Effect of sunlight treatment on the differential coloration of orchard-based apple fruit ‘Summer Prince’ (**A**). The fruits showed different coloration patterns between the three groups: reflected sunlight, bagged, and control (**B**) which is indicated in the color index (**C**) after seven days of treatment. Different letters above the bars indicate a significant difference (*p* < 0.05). Data are presented as mean ± SD (*n* = 20) of three biological replicates.

In this study, to elucidate the effect of sunlight on the coloration of apple peel, experiments were conducted on two commercial red-peel apple cultivars named ‘Arisoo’ (Figure 1A), and ‘Summer Prince’ (Figure 2A), under field conditions. The different sunlight treatments resulted in different coloration patterns between the three groups in two apple cultivars. Fruit coloration patterns showed positive correlation with light levels. Fruit in the bagged group (BA) exhibited colorless, increase in the control group (CT) and showed the highest coloration levels in the reflected group (RE). In the BA, the fruits were red-colorless in the dark condition, and a similarity between the exposed side (ES) and shaded side (SS) of the fruit was observed. In the CT, the ES was redder than the SS. In the RE, both sides of the fruit exhibited red color with a similar coloration pattern (Figure 1B and Figure 2B). This result shows the vital influence of sunlight on the different colorations in apple fruit. There were different coloration patterns between the two sides of the same fruit. The side exposed to direct sunlight was redder than the SS, obscured by a tree canopy (in CT). However, after exposure to reflected sunlight, the SS showing an absence of red color looked similar to the ES/RE.

Color index (CI) showed positively correlated with the red-coloration level. There was no significant difference in CI value between the ES and SS of the fruit in each independent BA and RE for both ‘Summer Prince’ and ‘Arisoo’. Significant differences between the two sides of the fruits were observed only in the CT with the CI value on the ES being higher than that on the SS. The CI value on both sides of the BA also did not show a significant difference from the CI on the SS/CT. The CI value in the RE was not significantly different between the two sides but significantly different for the ES/CT. In ‘Arisoo’, the CI also showed similarities between the two sides in the RE but was slightly lower than that in the ES of the CT (Figure 1C). ‘Summer Prince’ showed a similar CI for both sides in the RE but a higher CI than that in the ES/CT (Figure 2C).

There were apparent differences in the coloration patterns across the three groups under different sunlight treatments. We observed a linear relationship between coloration and sunlight intensity. When solar irradiation was blocked, the fruits in the bagged group showed an absence of the red color, with a lower CI value. In contrast, with enhanced solar irradiation by reflected sunlight, the SS of fruits with less color rapidly turned red. The crucial role of light in stimulating coloration in apple fruits has been illuminated in other studies. After removing the bag from the bagged apple fruit for re-exposure to sunlight, it quickly turned red [40], upgrading the contents of anthocyanin compounds [41,42,43].

### 2.2. Expression Profiles of the Carotenoid Biosynthesis-Related Genes under Light Treatment

**Figure 3:**
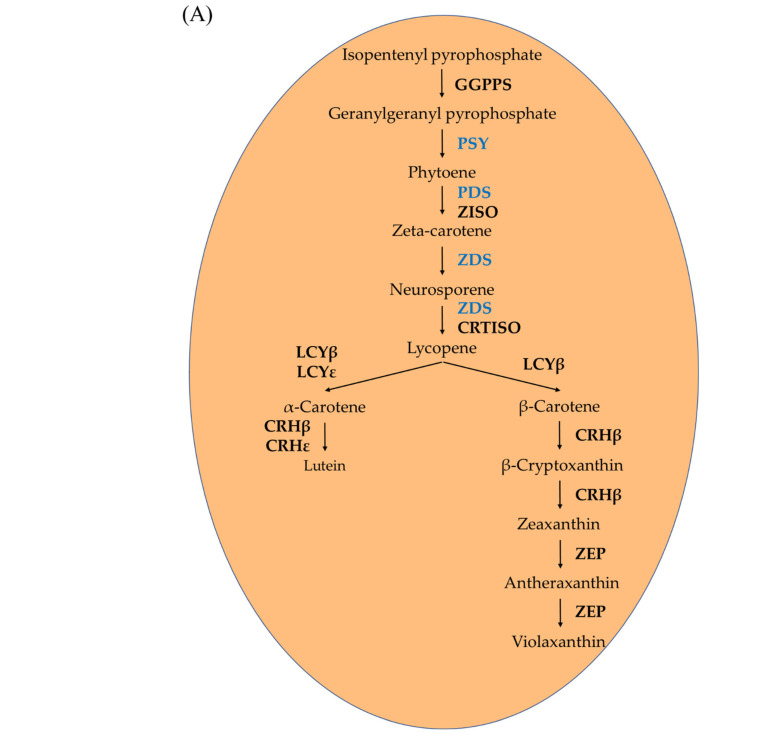

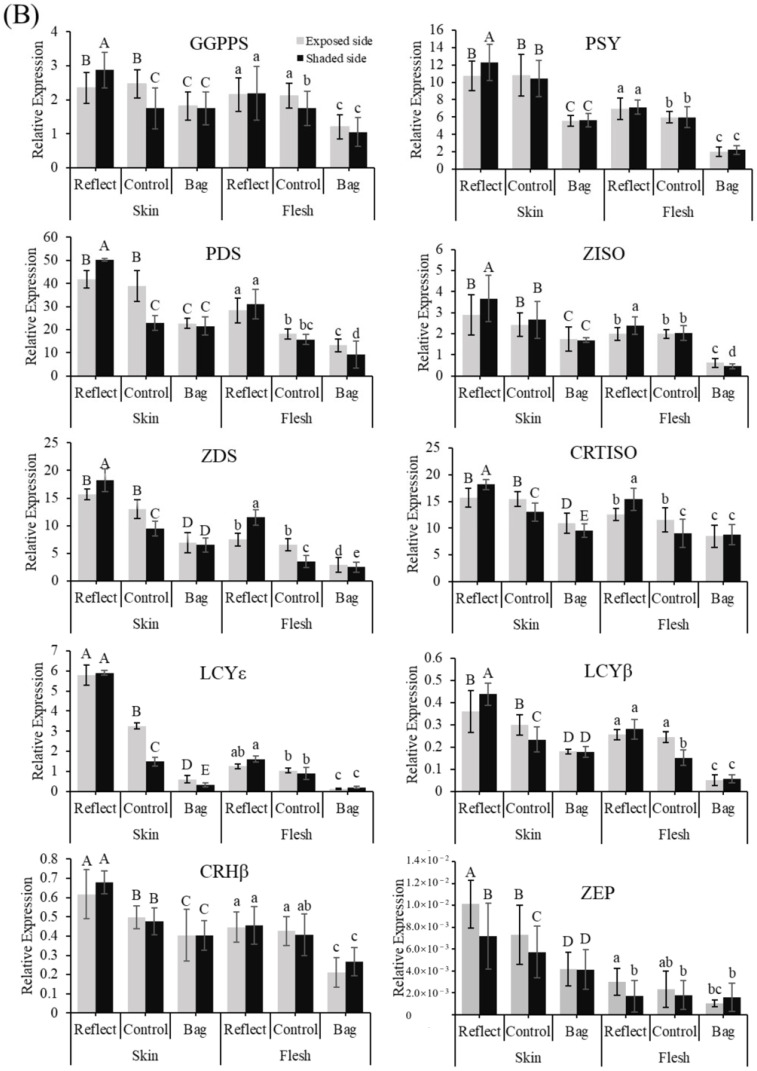

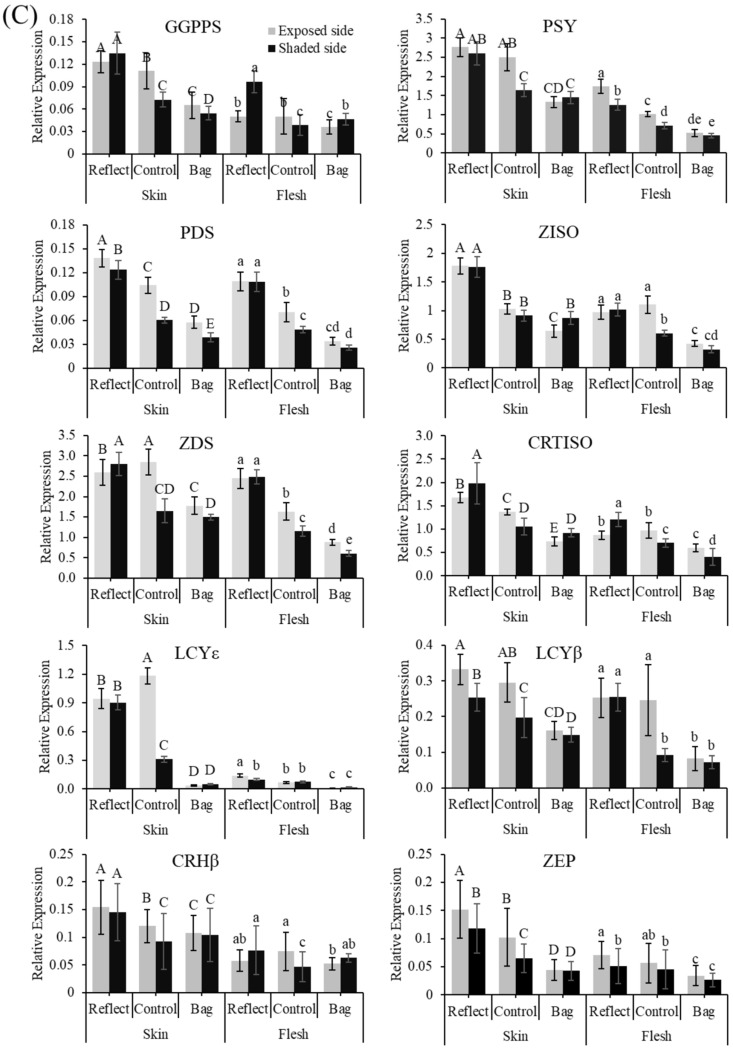
Carotenoid biosynthesis pathway (**A**). Expression profiles of carotenoid biosynthesis-related genes in the peel and flesh of three apple fruit groups: reflected, control, and bagged on the exposed and shaded sides of the apple ‘Arisoo’ (**B**), and ‘Summer Prince’ (**C**) under different treatment conditions. All qRT-PCR reactions were normalized to an apple SGF 29 tudor-like domain-containing protein (*MDP0000336547*) gene. Different uppercase and lowercase letters represent a significant difference (*p* < 0.05) in the peel and flesh, repectively. Data are means ± SD of three biological replicates.

To evaluate the effect of sunlight on different coloration patterns of apple fruits, we then quantified the transcript levels of ten carotenoid biosynthesis-related genes in the carotenoid biosynthetic pathway (Figure 3A) by qRT-PCR (Figure 3B,C). Concurrent with the coloration pattern, the expression levels of carotenoid biosynthesis-related genes showed a significant difference in the three groups, with a tendency to increase with sunlight intensity. The expression patterns of these genes were similar in both the apple cultivars.

In the BA and RE, the expression level of these genes was not significantly different between the ES and SS of fruits. However, significant differences between the two sides in the CT were observed. The expression of these genes on the shaded side was always lower than that of the exposed side, except *PSY*, *ZISO*, and *CRHβ* for ‘Arisoo’ (Figure 3B), and *ZISO* for ‘Summer Prince’ (Figure 3C). However, after exposure to sunlight, the expression level of these genes in the SS/RE was similar to the exposed side, upregulated to levels much higher than that in the control group (*LCYɛ* and *CRHβ* for ‘Arisoo’; *GGPPS*, *PSY*, *ZISO*, *LCYɛ*, and *CRHβ* for ‘Summer Prince’). Some genes such as *GGPPS*, *PSY*, *PDS*, *ZISO*, *ZDS*, *CRTISO*, and *LCYβ* even showed higher expression levels than the ES for ‘Arisoo’ (Figure 3B); *ZDS* and *CRTISO* were higher for ‘Summer Prince’ (Figure 3C).

### 2.3. Accumulation of Pigment Content under Light Treatment

**Figure 4:**
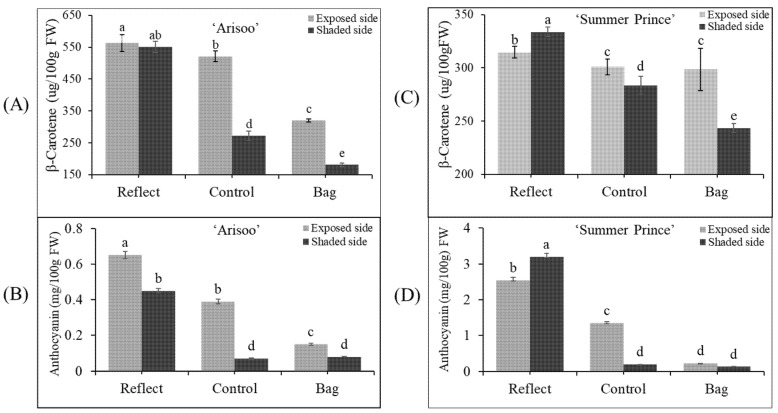
Accumulation of pigment content (β-carotene and anthocyanin) in the peel of three apple fruit groups: reflected, control, and bagged on the exposed side and shaded side of the apple ‘Arisoo’ (**A**,**B**) and ‘Summer Prince’ (**C**,**D**) under different treatment conditions. Different letters above the bars indicate a significant difference (*p* < 0.05). Data are the mean ± SD (*n* = 10) of three biological replicates.

After investigating the effect of light on the variation in coloration of apple fruits at the transcriptional level by qRT-PCR, we also evaluated the accumulation of pigment content to gauge its impact at the translational level. The accumulation of pigment content in the peel of apple fruits from the three groups is shown in Figure 4. Parallel to results obtained with the coloration pattern and the transcript levels, the accumulation of pigment content was significantly different in the three groups. The pigment content accumulated the highest in RE and lowest in BA.

The β-carotene content was the highest on the ES/RE for ‘Arisoo’ (563.17 μg/100 g FW) and on the SS/RE for ‘Summer Prince’ (333.79 μg/100 g FW). The accumulation was lowest in the SS/BA for both cultivars, with 180.86 μg/100 g for ‘Arisoo’ and 243.27 μg/100 g FW for ‘Summer Prince.’ In the CT, the β-carotene content on the SS was less than on the ES. However, in the RE, the accumulation of β-carotene reached similar levels to that of the exposed side for ‘Arisoo’ or even higher for ‘Summer Prince’. Another primary pigment, anthocyanin, also showed a similar trend with β-carotene content (Figure 4B,D). Taken together, the accumulation of pigment content was positively correlated with the intensity of sunlight.

When comparing the two cultivars, there was a significant difference in the accumulation of pigment content. The accumulation of β-carotene in ‘Arisoo’ was higher than in ‘Summer Prince.’ However, the anthocyanin content in ‘Arisoo’ was less than in ‘Summer Prince.’ These differences were observed at the transcript levels of genes between the two cultivars (Figure 3B,C). Therefore, these results indicate that this difference is likely to be controlled by genetic factors. In other studies, it was found that the bagging treatment may cause a reduction in the metabolism of carbohydrates [44,45]. As carbohydrates are required substrates for the synthesis of phenolic compounds, bagging treatment could affect the phenolic synthesis in apple peel via the regulation of carbohydrate metabolism [41]. These results showed that the level of pigment content depends on the expression level of the genes involved in their biosynthesis.

### 2.4. Construction of Expression Vectors

**Figure 5:**
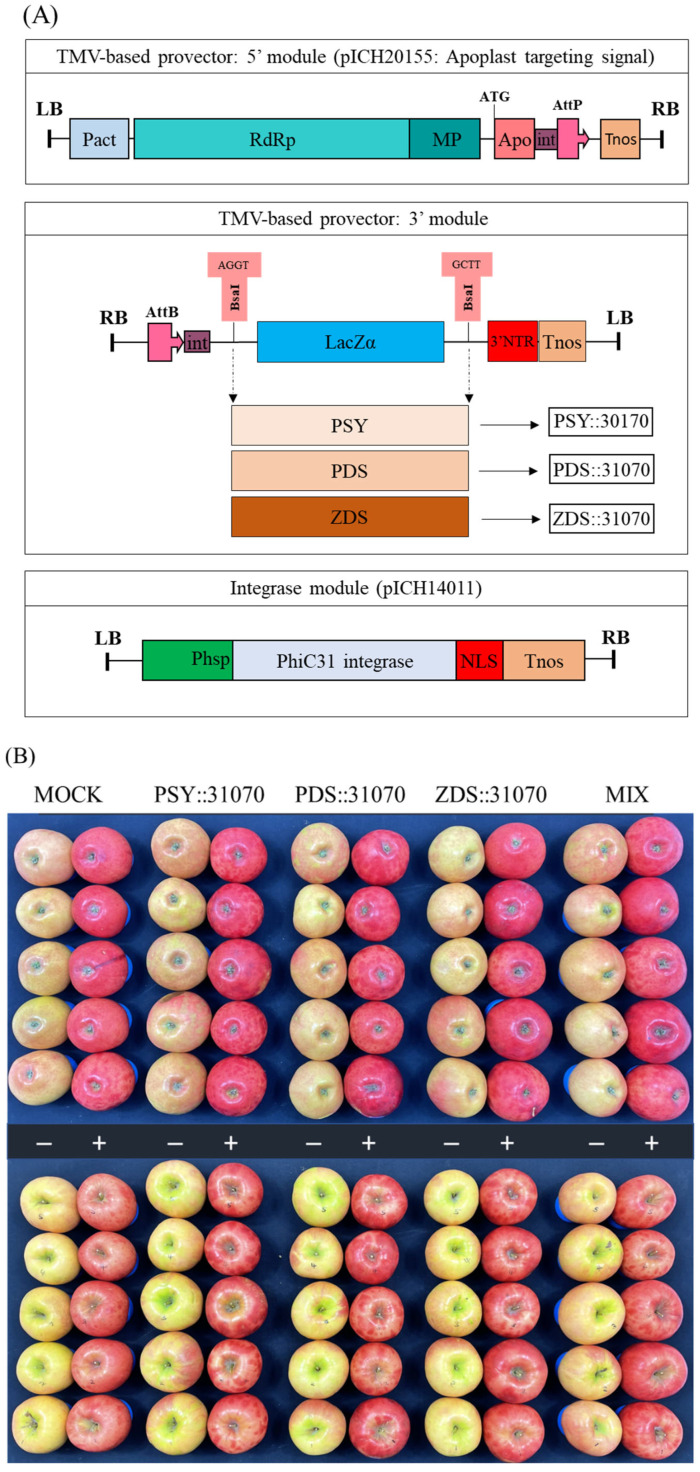
Schematic diagram of expression vectors for three carotenoid biosynthesis-related genes PSY::301070, PDS::31070, and ZDS::31070 (**A**). Apple ‘RubyS’ fruits were Agro-infiltrated with the expression vectors containing the pro-vector parts (5′ and 3′) and the integrase source using a vacuum after seven days of treatment with darkness (−) and light exposure (+) (**B**).

The genes MdPSY, MdPDS, and MdZDS were cloned into a plant expression vector pICH31170, yielding expression vectors PSY::31070, PDS::31070, and ZDS::31070, respectively (Figure 5A). The pICH31070 vector is a TMV-based 3′ pro-vector module for cloning these three genes. pICH20155 is a TMV-based 5′ pro-vector that targets the expressed genes to apoplasts due to the translational fusion with an apoplast-targeting signal peptide from rice alpha-amylase. pICH14011 is a binary vector for the expression of PhiC31 integrase, which provides the recombination between 5′ and 3′ pro-vectors resulting in a complete (functional) viral vector. The cloning of the PSY::31070, PDS::31070, and ZDS::31070 expression vectors is shown in Figure S2. The plant expression vectors were transformed into *Agrobacterium tumefaciens* EHA105; the ‘RubyS’ apple fruits were vacuum-infiltrated with the successful *A. tumefaciens* EHA105 transformants harboring the recombinant vectors. In this study, three carotenogenic genes were transiently expressed in apple fruit using the plant virus-based expression systems. This vector system can rapidly produce a high level of proteins due to the replication of the mRNA virus [46].

### 2.5. Effect of Light Treatment and Transient Expression of Carotenogenic Genes on Apple Fruit Coloration

**Figure 6:**
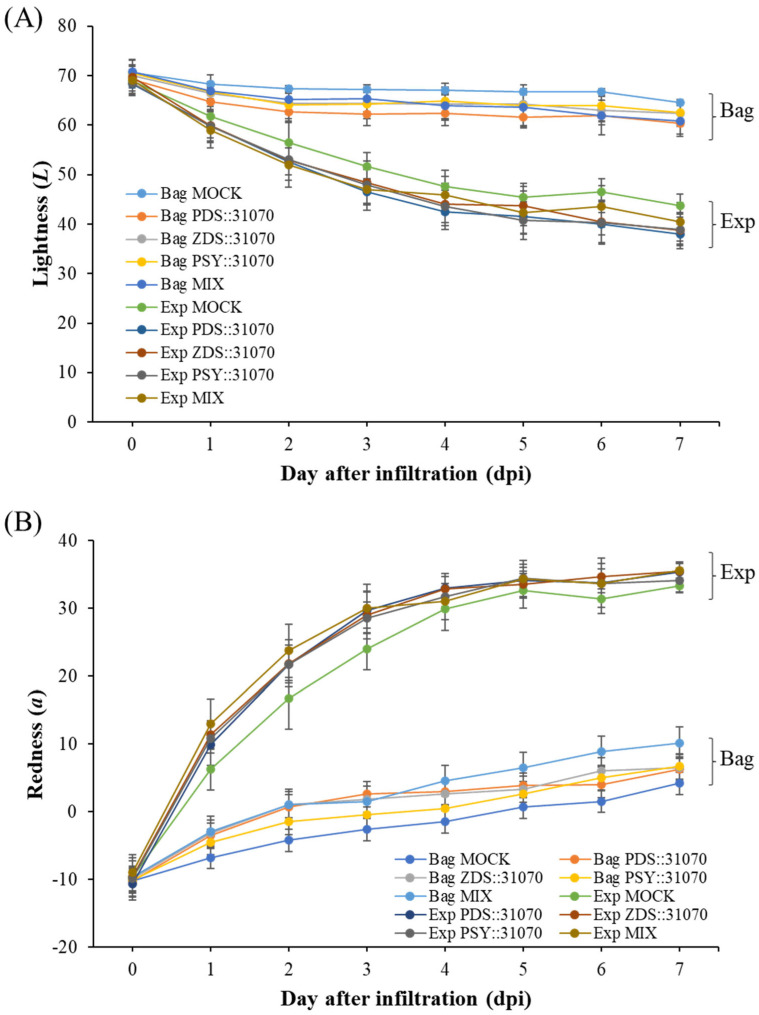

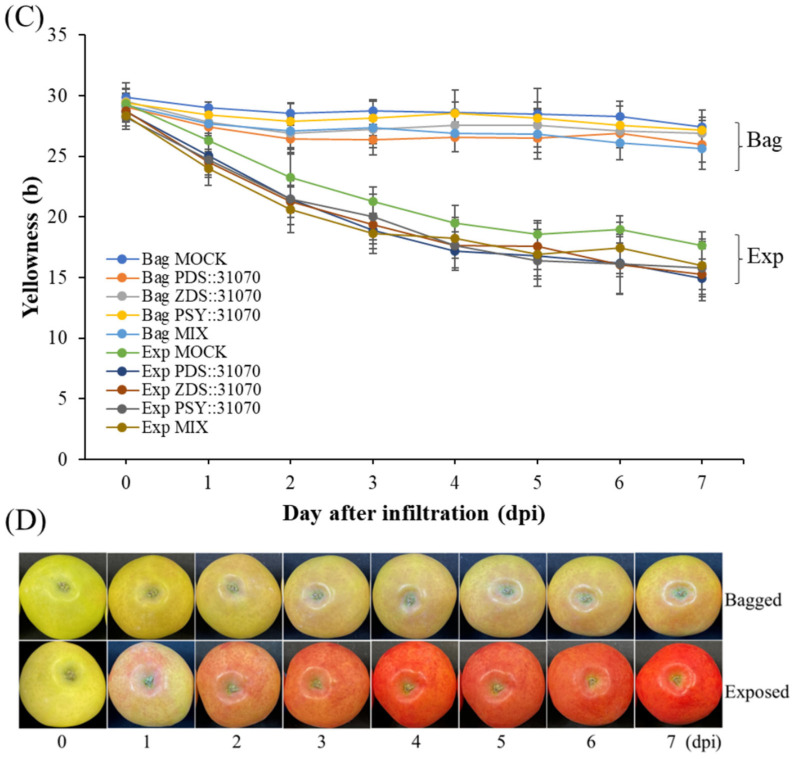
Skin color properties *L* (**A**), *a* (**B**), *b* (**C**) of ‘RubyS’ apple fruit under light treatment and overexpression of carotenoid biosynthesis-related genes. Agro-vacuum infiltrated fruits with a mixture of three expression vectors, PSY::301070, PDS::31070, and ZDS::31070, showed a different coloration pattern between the bagged and exposed fruits (**D**). Data are presented as mean ± SD (*n* = 12). *L*: lightness; *a*: red/green value; *b*: blue/yellow value.

After vacuum-infiltration, the ‘RubyS’ apple fruit was subjected to varying light treatments (darkness and exposure). The infiltrated fruits after seven days of light treatment are shown in Figure 5B. The results showed many significantly different coloration patterns among the bagged (−) and exposed (+) fruits, and a slightly different coloration pattern was observed in the five groups. The variable coloration of infiltrated fruits was recorded for seven days at one-day intervals; the coloration level increased during the light treatment (Figure 6). Over time, changes in color values were substantial during the treatment, especially on the exposed fruit. *L* (Figure 6A) and *b* (Figure 6C) continuously decreased, while *a* (Figure 6B) value increased. In the CT (mock), the color variables (*L*, *b*) showed a higher value in the bagged fruit than in the exposed fruit. In contrast, redness (*a*) in the CT showed a lower value than in the fruit group infiltrated with PSY::31070, PDS::31070, and ZDS::31070. The differences in color variation were not significantly different at the beginning of the treatment (Table S2) and then showed a tendency to increase day by day during the light treatment (Figure 6D). Regardless of the light treatments, the *a* value was consistently positively correlated with the light intensity level, while *L* and *b* values were negatively correlated with light intensity. The color variation also correlated with the gene expression treatments, but the changes were not as significant as the light factor. The effect of light on the level of fruit coloration is more drastic than the transient expression of these genes. These results indicate that environmental factors likely control this difference, showing the crucial role of light in determining fruit color.

### 2.6. Expression Profiles of the Carotenogenic Genes under Light Treatment and Transient Expression

**Figure 7:**
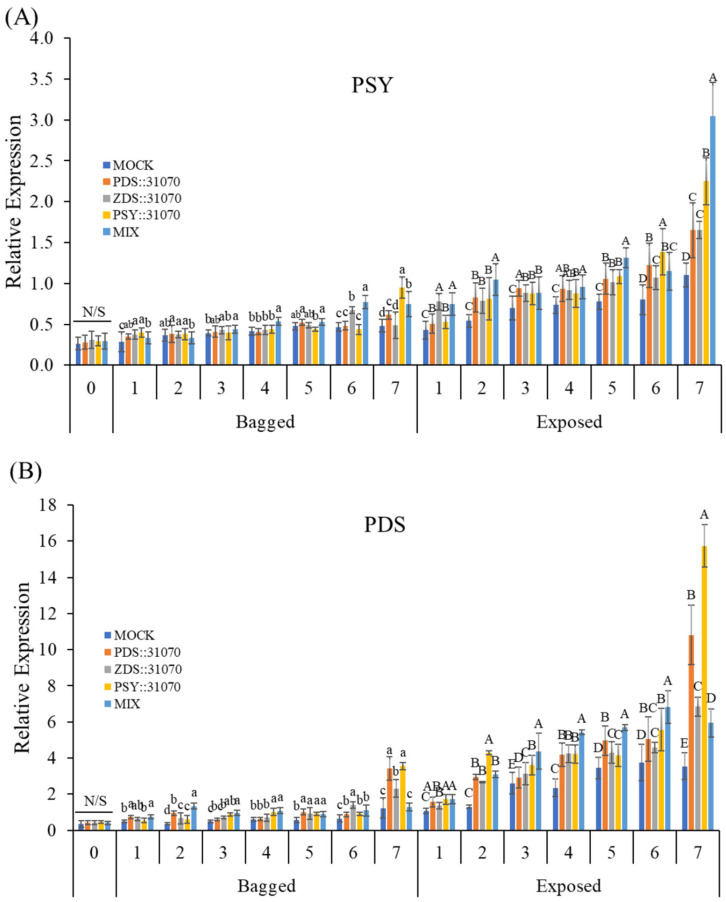

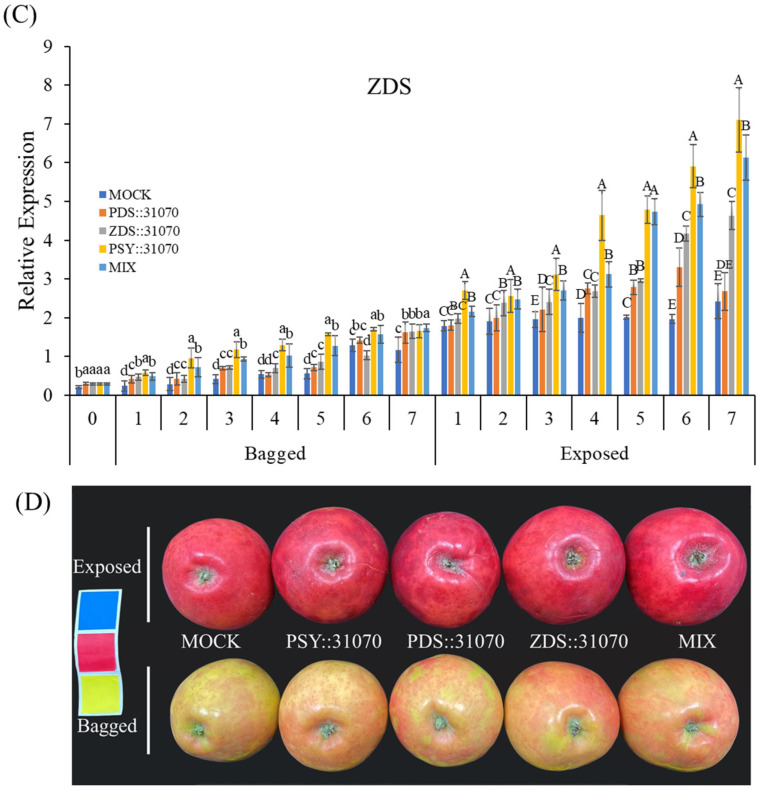
Expression profiles of carotenoid biosynthesis-related genes PSY (**A**), PDS (**B**), and ZDS (**C**) in darkness (bagged) and after light exposure (exposed) for the ‘RubyS’ apple fruit skin after Agro-vacuum infiltration. Agroinfiltrated fruits at 7 dpi (**D**). MOCK fruits were Agro-vacuum infiltrated with an empty vector; PSY::31070, PDS::31070, and ZDS::31070 fruits were Agro-vacuum infiltrated with a single expression vector, respectively; MIX fruits were Agro-vacuum infiltrated with a mixture of three (PSY::31070, PDS::31070, and ZDS::31070) expression vectors. Different lowercase and uppercase letters represent a significant difference (*p* < 0.05) in the bagged and exposed fruits, repectively. N/S refers to there being no significant difference (*p* < 0.05) among groups. Data are presented as mean ± SD (*n = 12)* of three biological replicates.

The transcript levels of three carotenogenic biomarker genes (*PSY*, *PDS*, and *ZDS*) were investigated by qRT-PCR in the peel of ‘RubyS’ apple fruits after agroinfiltration and light treatments (Figure 7). The expression levels of these genes in the fruits under light exposure were higher than that in the bagged fruits. Both exposed or bagged treatments tended to increase expression from 1–6 days post-infiltration (dpi) and then reach the peak at 7 dpi in five groups for all three carotenogenic biomarker genes (Figure 7A–C). In the bagged treatment, their expression level gradually increased. However, the increase in expression was steep upon light exposure, and many significant differences were observed. Moreover, under the same conditions of light treatment, the expression of carotenogenic biomarker genes was significantly different between the five groups, especially for *ZDS* (Figure 7C). Their expression in agroinfiltrated fruits was higher than in the mock. Regardless of gene expression, the differences in coloration patterns were significantly different between the light treatments (Figure 7D). Interestingly, the fruit agroinfiltrated with the PSY::31070 showed the highest expression level among all three carotenogenic biomarker genes investigated. This result could be because *PSY* is the first gene in the non-MEP pathway encoding the enzyme phytoene synthase catalyzing the substrate GGPP before feeding into the carotenoid biosynthesis pathway. *PSY* likely plays the role of a gatekeeper in this case. The function of *PSY* genes in response to abiotic factors and post-transcriptional feedback regulation has been well worked out and accepted as the most important gene in the carotenoid biosynthesis pathway [8]. These results show that light, combined with the transient expression of carotenogenic genes, upregulates the expression of the carotenoid biosynthesis-related genes.

### 2.7. Accumulation of Pigment Content under Light Treatment and Transient Expression

**Figure 8:**
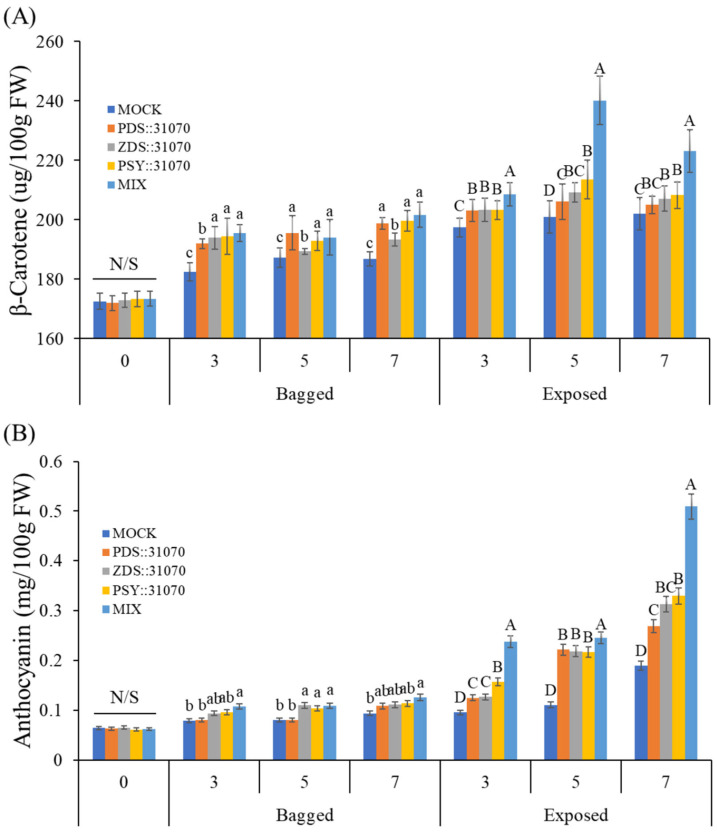
Accumulation of β-carotene (**A**) and anthocyanin (**B**) in the skin of the apple ‘RubyS’ under different treatment (light and overexpression of carotenoid biosynthesis-related genes) conditions. Different lowercase and uppercase letters represent a significant difference (*p* < 0.05) in the bagged and exposed fruit. N/S refers to there being no significant difference (*p* < 0.05) among groups. Data are presented as mean ± SD (*n* = 12) of three biological replicates.

After investigation at the transcriptional level by qRT-PCR, the accumulation of pigment contents was analyzed to clarify the effect of light in combination with transient expression of carotenogenic genes on the different colorations of apple fruits. The accumulation of pigment contents in the peel of ‘RubyS’ apple fruits from the five groups is shown in Figure 8. The accumulation of β-carotene in the exposed fruits was much higher than in the bagged fruits. Before the treatment, the β-carotene amounts were not significantly different among the five groups, with a value of 173 (µg/100 g FW). However, the β-carotene accumulation increased after 3–7 dpi. In the agroinfiltrated fruits with transient expression of carotenogenic genes also, β-carotene accumulation was higher than in the mock. In the exposed fruits, the accumulation of β-carotene increased at 3 dpi, reaching the most elevated amount at 5 dpi in the MIX group (a group with a mixture of the three expression vectors) at a value of 240 (µg/100 g FW), and then slightly decreased at 7 dpi (Figure 8A). Additionally, the content of other carotenoids such as α-carotene and other products downstream of the carotenoid biosynthetic pathway also showed similar phenomena as the accumulation of β-carotene (Table S3).

The content of another major pigment, anthocyanin, was also analyzed (Figure 8B). Similar to the β-carotene, anthocyanin accumulation in the exposed fruits was much higher than in the bagged fruits. In the bagged fruits, anthocyanin amounts did not increase much after treatment but showed a considerable increase in the exposed fruit. The anthocyanin accumulation in exposed fruits showed a significant difference among the five groups. It also showed the highest value of 0.51 (mg/100 g FW) for a mixture of the 3 vectors and 0.19 (mg/100 g FW) for the mock.

In a comparison of β-carotene and anthocyanin among the five groups in the darkness treatment (bagged fruits), there were many significant differences in the accumulation of β-carotene but not anthocyanin. The difference arises because more β-carotene can be produced from the transient expression of vectors harboring carotenogenic genes in the agroinfiltrated fruits despite being in the dark. Light exposure (exposed fruits) led to the accumulation of both β-carotene and anthocyanin. In this case, the accumulation of anthocyanin was regulated by light. Light exposure and transient expression of carotenogenic genes increase the accumulation of β-carotene.

Exposure to light increased not only the accumulation of β-carotene but also anthocyanin. In this study, we find that the transient expression of carotenogenic genes altered the anthocyanin accumulation, and there is a positive correlation between carotenoid and anthocyanin. In contrast, another study overexpressing CrtB protein on citrus embryogenic calli showed that carotenoids have a negative effect on anthocyanin accumulation [47]. It is difficult to define a causal relationship between carotenoid and anthocyanin accumulation when carotenogenic genes or anthocyanin biosynthetic genes are overexpressed. However, in nature, carotenoids and anthocyanins are often involved in concurrent biosynthetic processes and can co-exist in plant tissues. Again, supported by data shown in Figure 4, there is a positive correlation between carotenoid and anthocyanin in both the apple cultivars. In this case, the causal relationship may depend on species, tissue type, and the developmental stage of the tissue.

### 2.8. Accumulation of Small Heat Shock Proteins (smHSPs) in Response to Heat Stress

**Figure 9:**
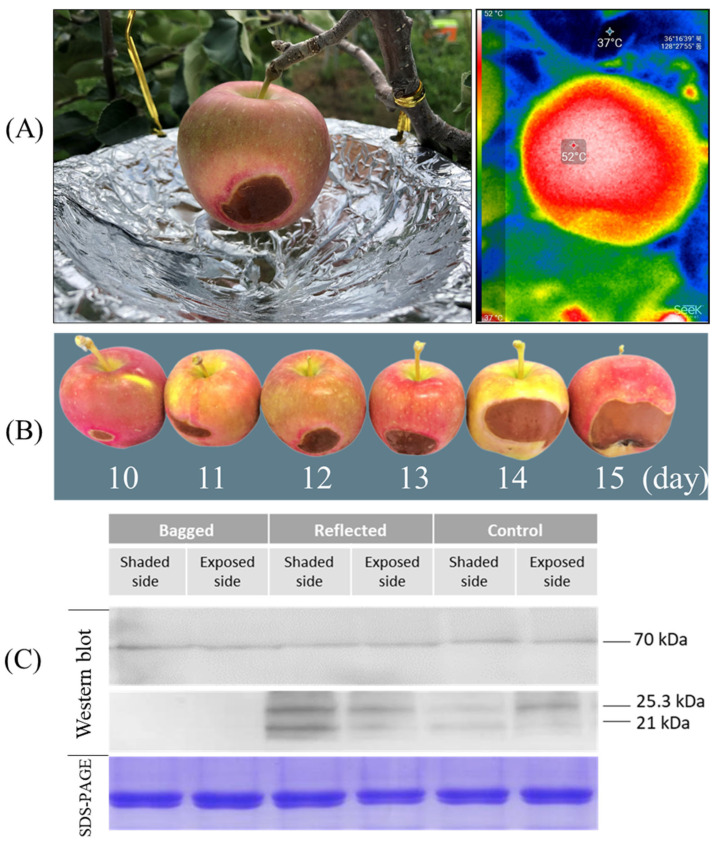
Symptoms of sunburn were observed on the shaded side in the fruit of ‘reflected sunlight’ where the fruit surface temperatures reached up to 52 °C in the hottest area, detected by thermal imaging (**A**). The sunburn damage was observed after ten days of sunlight treatment, and the damage level increased with increasing time of exposure (**B**). Immunoblotting assays (western blot) were carried out on the apple ‘Arisoo’ to evaluate the accumulation of smHSP in response to thermal stress with Coomassie Brilliant Blue-stained (SDS-PAGE) protein serving as a loading control (**C**).

In the experiment conducted, we observed symptoms of sunburn in fruits treated with reflected sunlight. Overheating due to solar radiation, air temperature, and radiative heat transfer (temperature produced from reflected sunlight with paper-wrapped aluminum disc) is the leading cause of sunburn. Thermal imaging showed that fruit surface temperatures reach up to 52 °C in the hottest areas exposed to reflected sunlight. The lowest temperature of 37 °C was recorded in the canopy area covered by leaves (Figure 9A). Exposure to reflected sunlight raises the temperature to 15 °C higher than tree canopy branches. In other fruit, such as berries, solar heat on the sun-exposed side increases fruit surface temperature up to 12–15 °C higher than air temperatures [48]. The level of damage caused due to sunburn increases parallelly with increasing exposure time of fruits to reflected sunlight. After ten days of treatment with reflected sunlight, sunburn damage begins on the fruit’s ES in the area that receives most of the reflected light. The sunburn damage got worse day by day with the increase of the damaged area getting bigger and bigger. On day fifteen, the size of the damaged area reached up to around 15% of the fruit’s surface (Figure 9B). In contrast, no sunburn damage was observed in fruits in the ‘control’ and ‘bagged’ group (data not shown). Therefore, bagging fruit in paper bags can block direct sunlight, thereby reducing the temperature of fruit inside the bag and helping prevent or reduce sunburn damage [49].

A western blot analysis was performed to compare the expression level of HSPs between three treatment groups in response to heat stress (Figure 9C). After ten days of sunlight treatment, the ‘Arisoo’ apple fruit was collected to extract total soluble protein (TSP), and subsequent immunoblotting was performed to detect the accumulation of HSPs. The expression level of HSP70 was constant under different sunlight treatments. However, the expression level of smHSPs (HSP25.3- and HSP21) was significantly different between the three groups. There are no signals of smHSPs detected in the ‘bagged’ group on both SS and ES. However, the expression of smHSPs shows that strong signals were detected in CT and RE. The expression level of smHSPs in the RE was higher than in the CT. In the CT, the ES showed higher expression than the SS, similar to the ES/RE. However, in the RE, the expression level of smHSPs for the SS was higher than that for the ES since fruits on the SS received more heat stress produced by reflection from the aluminum-wrapped disc. Results indicated that the accumulation of smHSP depends on the degree of thermal stress, and the increase is positively correlated with the level of sun exposure. The accumulation of HSP70 did not show variation, while smHSPs showed significant differences under different sunlight treatments. This difference may be because smHSPs are more sensitive and most abundant expressed among many HSPs when subjected to thermal stress [39]. Another study in pea (*Pisum sativum*) showed that upon heat stress, the amount of smHSPs increases up to 1000 to 2000-fold [50].

## 3. Materials and Methods

### 3.1. Fruit Materials and Sunlight Treatments

The fruit materials and sunlight treatments were conducted as described previously [35]. Briefly, the fruit of two apple cultivars, ‘Summer Prince’ and ‘Arisoo’ at 60 days after full bloom were divided into three groups (bagged, reflected, and control) regarding different sunlight treatments. The fruits of each cultivar were divided into three treatment groups: ‘control’, ‘bagged’, and ‘reflected sunlight’ groups. In the first group, fruits were grown under normal sunlight conditions as a control. In the second group, the fruits were bagged with double-layered paper bags. In the third group, the fruits were exposed to sunlight by a paper wrapped-aluminum disc. After seven days of treatment, in each cultivar, forty fruits in each group were randomly collected from several trees. Twenty fruits were used to measure fruit color properties and fruit characteristics. The other ten fruits were used for RNA isolation. The peel of the remaining ten fruits was used for pigment analysis. Samples for RNA isolation and pigment content analysis were immediately frozen in liquid nitrogen and stored at −80 °C after sampling.

### 3.2. Measurements of Fruit Color and Fruit Characteristics

Variations in the peel color of fruits were measured using a chromameter (CR-400, Konica Minolta, Tokyo, Japan). Fruit firmness was measured using a digital fruit firmness tester (TR Turoni, Forlì FC, Italy). Soluble solids content used a pocket refractometer (PAL-1, Atago, Japan). Titratable acidity titrated using 0.1 N NaOH, expressed as % malic acid equivalents. CI were calculated as previously reported [35].

### 3.3. Construction of Plant Expression Vectors and Agrobacterium Transformation

The 1st strand cDNA was synthesized from total RNA isolated from the peel tissues of ‘Arisoo’ apple cultivars by reverse transcription PCR according to the manufacturer’s instructions (PrimeScript™ 1st strand cDNA Synthesis Kit, Cat. #6110A, Takara, Kusatsu, Japan) using specific primers. Specific primers for PCR amplification were designed to contain a BsaI restriction enzyme site at the 5′ end of the forward and reverse primers (Table S1) to clone *MdPSY* (GenBank: KT189149.1), *MdPDS* (GenBank: KU508828.1), and *MdZDS* (GenBank: AF429983.1) into an expression vector. CDS of three carotenoid biosynthesis-related genes were shown in Figure S1. PCR products were analyzed on 0.8% agarose gels, and the target band of these genes was recovered from gels and purified using a fragmented DNA purification kit (iNtRON MEGAquick-spin™ Plus, Seongnam, Korea). The fragment was then ligated into the pGEM^®^-T Easy vector (Promega, Madison, WI, USA), transformed into *E. coli* DH5α competent cells (Cat. #9057, Takara, Kusatsu, Japan), and the plasmid DNA was sequenced (Macrogen, Seoul, Korea). The accuracy of the DNA sequencing for *MdPSY*, *MdPDS*, and *MdZDS* was confirmed using CLC Genomic Workbench 12. The *MdPSY*, *MdPDS*, and *MdZDS* genes were digested with BsaI restriction enzyme and then cloned into an expression vector pICH31070 which was also digested by the same restriction enzyme to produce the recombinant expression vectors.

For the transient expression assay, the expression vectors harboring *MdPSY*, *MdPDS*, and *MdZDS* genes were transformed into *A. tumefaciens* EHA105 using the freeze-thaw method [51]. After transformation, *A. tumefaciens* EHA105 was spread on an LB agar medium containing antibiotics (50 μg/mL kanamycin and 100 μg/mL rifampicin) and cultured for two days. The transformed A. tumefaciens EHA105 colonies were chosen and inoculated in 5 mL each of liquid LB medium containing antibiotics and grown at 28 °C with shaking at 180 rpm in dark conditions overnight. Plasmid DNA of transformed *A. tumefaciens* EHA105 was isolated and then confirmed for the presence of the target genes in the expression vector by enzyme digestion.

### 3.4. Agrobacterium Vacuum-Infiltration

For the functional study of *MdPSY*, *MdPDS*, and *MdZDS*, they were transiently expressed in the ‘RubyS’ apple fruit using *Agrobacterium* vacuum-infiltration. The transformed *Agrobacterium* was initially cultured in 5 mL LB. The next day, one milliliter of the culture was transferred to a 250 mL flask containing 50 mL of LB broth medium supplemented with antibiotics. The cell culture grown overnight was centrifuged at 4000× *g* for 15 min to collect the *Agrobacterium* cells. The supernatant was removed, and the cell pellet was suspended in an infiltration buffer (10 mM MgCl_2_, 10 mM MES, pH 5.5, and 200 μM acetosyringone) to reach a final OD_600_ of 0.5. The Agrobacterial suspension was then maintained in the dark for 2–3 h at room temperature before infiltration.

The transformed *A. tumefaciens* EHA105 suspensions harboring the individual vectors were vacuum-infiltrated into the ‘RubyS’ apple fruit. The ‘RubyS’ fruits were submerged in the agrobacteria/infiltration buffer and vacuumed at 500 mmHg for 2 min (Figure S3). The infiltrated fruits were divided into five groups: (1) mock treatment/control (empty vector), (2) PSY::31070, (3) PDS::31070, (4) ZDS::31070, and (5) MIX (a mixture of three vectors (2), (3), and (4)) with 120 fruits per group. After the *Agrobacterium* vacuum-infiltration, the fruits were blotted in tissue paper to remove the *Agrobacterium* residues on the outer surface of the fruits and then kept in darkness overnight. The next day, the infiltrated fruits of the five groups were treated with LED light with the 16-h light/8-h dark photoperiod or kept in darkness at 25 °C. In each group, twelve fruits were collected at one-day intervals during the light treatment for fruit color indices and then peeled for RNA isolation. Twelve other fruits were collected on the third, fifth, and seventh days for pigment content analysis.

### 3.5. RNA Extraction and Quantification of Gene Expression

Total RNA was isolated from peel and flesh (‘Summer Prince’ and ‘Arisoo’) and peel (‘RubyS’) using the CTAB method [52]. Then removing genomic DNA in the RNA samples by treating with DNase (TURBO DNA-free™ Kit, Cat. #AM1907, Invitrogen, Carlsbad, CA, USA). Evaluate their concentration and quality using a UV spectrophotometer (BioDrop µLite, Biochrom, Cambridge, UK). 1.0 μg of total RNA was used as a template for first-strand cDNA synthesis with oligo dT primer (PrimeScript™ 1st strand cDNA Synthesis Kit, Takara, Kusatsu, Japan).

Gene expression was analyzed using qRT-PCR on a LightCycler 480 II Real-Time PCR System (Roche Diagnostics, Mannheim, Germany). qRT-PCR procedure was conducted with the LightCycler 480 SYBR Green I Master Mix (Roche, Basel, Switzerland) as described previously [35]. Primers were designed to obtain a 100–150 bp product using Primer-Blast (http://www.ncbi.nlm.nih.gov/tools/Primer-Blast (accessed on 10 September 2021). Relative expression levels of target genes were normalized to a reference gene *MDP0000336547* [53]. The raw data were analyzed with Light Cycler^®^ 480 Software release (version 1.5.1, Roche, Basel, Switzerland). The primer sequences for the qRT-PCR are listed in Table S2.

### 3.6. Pigment Content Analysis

#### 3.6.1. Carotenoid Analysis

Frozen apple peel samples (~150 mg) were homogenized to a fine powder using a mortar and pestle. 3% pyrogallol and 60% KOH were added to a certain amount of sample, saponified in a water bath at 70 °C, and then cooled. After adding a 1% NaCl solution and a hexane and ethyl acetate mixture, the samples were homogenized by vortexing. The supernatant was collected by centrifugation. Again, a hexane and ethyl acetate mixture solution was added to the extracted supernatant. The supernatant was re-extracted following centrifugation. This process was repeated until all the pigment was gone. The collected supernatant was entirely concentrated with nitrogen gas and then dissolved in ethanol and used as the sample for carotenoids analyzed using HPLC (Shiseido SP LC SP3202, Tokyo, Japan).

A 10 μL aliquot was injected into the HPLC system with column Shiseido UG 120. Carotenoids were separated using a mobile phase (Acetonitrile: Methanol: Dichloromethane) at a flow rate of 1 mL/min, and the column temperature was maintained at 40 °C. Carotenoids were detected at a wavelength of 450 nm, and the concentrations of carotenoids were determined as β-carotene equivalents per g of fresh tissue weight. β-carotene was identified in the extracts by comparing retention times and online spectral data with standard samples.

#### 3.6.2. Anthocyanin Analysis

Total anthocyanin content was determined using the pH differential method [54]. Briefly, a certain amount of homogenized samples was added to 25 mL of methanol containing 1% HCl and shaken for 20 min at 4 °C in darkness. The homogenates were sonicated, and the supernatant was collected separately after centrifugation (4000 rpm, 10 min). A buffer solution of pH 1.0 and a buffer solution of pH 4.5 was added to a predetermined amount of the supernatant, stirred, and absorbance was measured at 520 nm and 700 nm. Anthocyanin content was expressed as cyanidin-3-glucoside equivalent; extinction coefficient was 26,900 L·cm^−1^mol^−1^ and molecular weight was 449.2 g·mol^−1^.

### 3.7. Heat Treatment and Protein Extraction

Fruits of the ‘Arisoo’ apple cultivar were treated with different solar irradiation (‘control,’ ‘bagged,’ and ‘reflected sunlight’) as described in part 3.1 to investigate the effect of heat generated from sunlight exposure. After ten days of sunlight treatment, symptoms of sunburn were observed on the exposed side of the fruits belonging to the ‘reflected sunlight’ group. The fruits from the three groups were picked from the tree at 3:00 pm and then held at 22 °C for 24 h before sampling. For small heat shock proteins (smHSPs) analysis, the peel of the ‘Arisoo’ apple cultivar was excised from the hottest area on the ES and the SS. Total soluble protein (TSP) was extracted using the method described by Ritenour with minor modifications [39].

### 3.8. SDS-PAGE and Western Blot Analysis

To characterize the expression of HSPs in apple peel after heat treatment and protein extraction, the concentration of TSP was determined by the Bradford protein assay (Cat. #5000207, Bio-Rad, Hercules, CA, USA) with bovine serum albumin as a standard. 30 μg of TSP for each sample was separated on a sodium dodecyl sulfate-polyacrylamide gel electrophoresis (SDS-PAGE) (Mini-protean TGX gels, Cat. #456-1093, Bio-Rad, Hercules, CA, USA) under reducing conditions. Proteins were separated in an electrophoresis buffer (25 mM Tris, 192 mM glycine, and 1% SDS) at 60 V for 30 min and then 120 V for 2 h using a mini-protean tetra system (Bio-Rad, Hercules, CA, USA). For western blot, proteins from the SDS-PAGE gel were transferred onto 0.45 µm nitrocellulose membranes (NC) (A23984263, GE Healthcare Life Sciences, Amersham, UK) in transfer buffer (50 mM Tris, 40 mM Glycine, and 20% methanol). After blotting the proteins to the NC, the membrane was blocked overnight with a blocking solution containing 5% non-fat milk in TBS-T buffer (60 mM Tris-HCl, pH 7.5, 1.5 M NaCl, and 0.05% Tween 20). The membrane was washed three times with TBS-T with a 5 min period between washes. Rabbit anti-heat shock protein 21 antibody (United States Biological, Salem, MA, USA) or rabbit anti-heat shock protein 70 antibody (LSBio, Seattle, WA, USA) was used as the primary antibody at a dilution of 1:5000 in the blocking solution for 2 h. After three 5-min washes in PBS-T buffer, goat anti-rabbit IgG (whole molecule) conjugated to alkaline phosphatase (Sigma-Aldrich, Louis, MO, USA) was used as the secondary antibody. The membranes were visualized following three 5-min washes in TBS using a premixed BCIP/NBT solution (B6404, Sigma-Aldrich, Louis, MO, USA).

### 3.9. Data Analysis

The results are expressed as means ± SD. Significant differences between multiple groups were determined by Tukey’s test. *p* < 0.05 indicates significant differences.

## 4. Conclusions

To investigate the role of light in the different coloration patterns of apple fruit, we analyzed the differential expression of carotenoid biosynthesis-related genes involved in controlling the development of coloration in the peel of the fruit of two red-skinned apple cultivars named ‘Arisoo’ and ‘Summer Prince.’ Further, to clarify the function of carotenoid biosynthesis-related genes in the different coloration patterns and pigment content accumulation of apple fruit, the *MdPSY*, *MdPDS*, and *MdZDS* genes were cloned and transiently expressed on the fruit of the apple ‘RubyS.’ Light, in conjunction with overexpression of these genes, upregulated carotenoid biosynthesis and accumulated pigment content. Light stimulates the coloration and pigment accumulation of apple fruits by enhancing the expression of carotenoid biosynthesis-related genes and the function of carotenogenic genes themselves. However, the effect of the light is more pronounced than that of intrinsic genetic factors. These findings further our understanding of the molecular mechanisms underlying different coloration and pigment accumulation in apple fruit, highlighting the important role of environmental stimulus and genetic factors in regulating gene expression. A better understanding of the genetic regulation of carotenoid biosynthesis and the effect of environmental stimuli can help design interventions in the carotenoid synthesis pathway to improve the color of apple fruits. In addition, sunburn symptoms in apple fruits were observed during the treatment of high intensity and long duration of light exposure. Whilst obtaining an improvement in fruit color using light irradiation, a warning that the appearance of sunburn caused by overheating due to light exposure should be considered.

## Data Availability

Not applicable.

## Supplementary Materials

The following supporting information can be downloaded at: https://www.mdpi.com/article/10.3390/ijms23116153/s1.
