# Supplementary material for: Light Induces Carotenoid Biosynthesis-Related Gene Expression, Accumulation of Pigment Content, and Expression of the Small Heat Shock Protein in Apple Fruit
Source: Int J Mol Sci. 2022 May 31;23(11):6153. doi: 10.3390/ijms23116153 (PMC9181450; doi:10.3390/ijms23116153)
Supplement: Supplementary file 1 [file ijms-23-06153-s001.zip › ijms-1725858-Supplementary.pdf]

Supplementary Materials

Table S1. Sequences of primers used in gene cloning and qRT-PCR

Table S2. Fruit characteristic and colorimetric coordinates of 'RubyS' before *Agrobacterium* vacuum-infiltration and light treatment

Table S3. Carotenoids content of 'RubyS' apples exposed to lights for 7 days.

Figure S1: DNA sequence (CDS) of three carotenoid biosynthesis-related genes cloned in this study

Figures S2–4. Gene cloning and *Agrobacterium* transformation of three carotenoid biosynthesis-related genes

Figure S5. *Agrobacterium* vacuum-infiltration in 'RubyS' apple fruit

Table S1. Sequences of primers used in gene cloning and qRT-PCR

Gene	Encoded protein		Primer sequence (5'-3')	Note
PSY	Phytoene Synthase	F	GGTCTCATGTCAGGTGTTCTTCTCTG	Primers used in cloning these genes into the expression vector. Blue: BsaI site Red: Start codon Purple: Stop codon
		R	GGTCTCTCATCTAGGCACCAACTGCTT	
PDS	Phytoene Desaturase	F	GGTCTCATGGCGCAGTGGGCTTGTGTCTCCGC	
		R	GGTCTCTCATCGAGCGGCTGCCTCAGCCAAC	
ZDS	Zeta-carotene Desaturase	F	GGTCTCATGGCTTCTTGGGCTCTTTTACCGGCA	
		R	GGTCTCTTAGACAAGACTCAGCTCATCAG	
GGPPS	Geranylgeranyl Pyrophosphate Synthase	F	TTCCTTGGCAACCCTGTCAC	Primers used in qRT-PCR
		R	ATGTCGACCGCCTCAAAGG	
PSY	Phytoene Synthase	F	GGACTGGAACTCACTCTTC	
		R	GCCGTAATCTGACAACAC	
PDS	Phytoene Desaturase	F	AGGATCACTAACGATCAGCTTCTCT	
		R	TGGCCAAACTTTGCTTTGCTG	
ZISO	Zeta-carotene Isomerase	F	CCAAGCAGTGGTGAACTC	
		R	CTTCTCTCCCTCCCTCAC	
ZDS	Zeta-carotene Desaturase	F	AAGCCTCATCACTGGACT	
		R	ATGTGAGAAAGGCAGAGG	
CRTISO	Carotenoid /prolycopene isomerase	F	GCTGGAGAAAGAAGTTGG	
		R	GGAGGATGAAGGTGAGTG	
LCYε	Epsilon lycopene Cyclase	F	CAACATCCAAGGTCTCTC	
		R	GTCCCATCTGAAGTAGCA	
LCYβ	Lycopene beta Cyclase	F	TCATCTCTCTACTGCAGTGCCTA	
		R	CCAGAAAGACACCTTAGACCTTCG	
CRHβ	B-carotene hydroxylase 2	F	CTTGAGTGTGATGAGAAAAGGTGC	
		R	CCTGTTTAAGCCTGCTTTGTATGG	
ZEP	Zeaxanthin Epoxidase	F	CGCCAGCATTTTCCCCAATTT	
		R	TCGAAATCTCGCAGCCCTTG	

Table S2. Fruit characteristic and colorimetric coordinates of 'RubyS' before *Agrobacterium* vacuum-infiltration and light treatment

**Group**	**Fruit characteristics**							**Colorimetric coordinates**		
	**Weight (g)**	**Vertical length (cm)**	**Horizontal length (cm)**	**Firmness (N)**	**SSC (ºBrix)**	**Acidity (%)**	**Starch index**	**L**	**a**	**b**
MOCK	74.3±5.4[a]	52.5±2.9[a]	56.2±3.4[a]	44.24±5.64[a]	12.2±0.51 [a]	1.45 [a]	1.6 [a]	68.87±2.01 [a]	-9.67±2.84 [a]	29.35±0.80 [a]
PYS::31070	75.0±4.6[a]	53.7±2.8[a]	56.6±2.5[a]	41.89±5.11[a]	12.4±0.67 [a]	1.50 [a]	1.5 [a]	68.30±1.93 [a]	-10.70±2.42 [a]	28.66±0.82 [a]
PDS::31070	73.6±5.1[a]	52.3±2.6[a]	56.4±2.9[a]	43.63±5.53[a]	12.5±0.35 [a]	1.50 [a]	1.6 [a]	69.68±2.13 [a]	-9.62±2.39 [a]	28.73±0.89 [a]
ZDS::31070	75.8±4.8[a]	53.0±2.9[a]	56.3±3.6[a]	42.65±5.23[a]	12.2±0.25 [a]	1.53 [a]	1.5 [a]	68.46±2.38 [a]	-9.90±2.69 [a]	28.26±1.01 [a]
MIX	74.5±4.3[a]	52.5±2.3[a]	56.3±2.3[a]	40.40±5.10[a]	12.1±0.15 [a]	1.53 [a]	1.5 [a]	69.13±3.11 [a]	-9.00±2.67 [a]	28.38±0.86 [a]

Data are means ± S.E. of three replications. Different letters above the bars indicate significant ($P < 0.05$) differences according to the t-test.

- L scale: Light vs. dark where a low number (0-50) indicates dark and a high number (51-100) indicates light.
- a scale: Red vs. green where a positive number indicates red and a negative number indicates green
- b scale: Yellow vs. blue where a positive number indicates yellow and a negative number indicates blue.

Table S3. Carotenoids content of 'RubyS' apples exposed to lights for 7 days.

Group	Time	Sample ID	α-Carotene	Capsanthin	Capsorubin	Zeaxanthin	β-Cryptoxanthin
Bagged	3dpi	MOCK	0.00	5.09e	Trace	0.00	0.00
		PYS::31070	0.00	14.65d	Trace	0.00	0.00
		PDS::31070	0.00	17.69c	Trace	0.00	0.00
		ZDS::31070	0.00	20.29b	Trace	0.00	0.00
		MIX	Trace	69.51a	Trace	0.00	Trace
	5dpi	MOCK	Trace	9.67e	Trace	0.00	Trace
		PYS::31070	Trace	43.48c	Trace	0.00	Trace
		PDS::31070	Trace	33.74d	Trace	0.00	Trace
		ZDS::31070	Trace	62.51b	Trace	0.00	Trace
		MIX	Trace	68.71a	Trace	0.00	Trace
	7dpi	MOCK	Trace	21.20d	Trace	0.00	Trace
		PYS::31070	Trace	61.29b	Trace	0.00	Trace
		PDS::31070	Trace	41.56c	Trace	0.00	Trace
		ZDS::31070	Trace	63.74b	Trace	0.00	Trace
		MIX	Trace	89.59a	Trace	0.00	Trace
Exposed	3dpi	MOCK	Trace	14.09e	Trace	0.00	Trace
		PYS::31070	Trace	19.19d	Trace	0.00	Trace
		PDS::31070	Trace	23.25c	Trace	0.00	Trace
		ZDS::31070	Trace	40.33b	Trace	0.00	Trace
		MIX	Trace	93.45a	Trace	0.00	Trace
	5dpi	MOCK	Trace	20.21e	Trace	0.00	Trace
		PYS::31070	Trace	39.25d	Trace	0.00	Trace
		PDS::31070	Trace	51.58c	Trace	0.00	Trace
		ZDS::31070	Trace	71.41b	Trace	0.00	Trace
		MIX	Trace	104.87a	Trace	0.00	Trace
	7dpi	MOCK	Trace	35.53e	Trace	0.00	Trace
		PYS::31070	Trace	43.88d	Trace	0.00	Trace
		PDS::31070	Trace	61.83c	Trace	0.00	Trace
		ZDS::31070	Trace	68.29b	Trace	0.00	Trace
		MIX	Trace	167.54a	Trace	0.00	Trace

The result marked as Trace means that a peak is detected but is below the limit of quantitation.

Figure S1. DNA sequence (CDS) of three carotenoid biosynthesis-related genes cloned in this study

1/ *Malus domestica* phytoene synthase (PSY) mRNA, complete cds
GenBank: KT189149.1
CDS: 1..1191

ATGTCAGGTGTTCTTCTCTGGGTGGTGAGTCCCAAAGAGAATGCCAGCTCTCCGCTGGGTCTGTTGC
CGAGAATTTGCACCCCAAGGAGGTCTAAATTGTGCTCAAAGCTGGGTTTTTCAAGTGGGGTTTTGGC
CTACTCGGGTGCAGTTGCAAACCCAGCCAGATCTTCAGAGGAGAAGGTGTATGAAGTGGTGCTGAAG
CAGGCTGCTCTAGTGAGAGAACCGAACACGGTAAAAAAGAAATCTTTGGATTTGGATGAACGGATTA
CTGAAGGTTTGAACAACTGGGATTTACTGAATAAGGCGTATGACCGGTGTGGTGAGGTCTGTGCAGA
GTATGCCAAGACTTTTTACCTAGGGACATTGCTCATGACACCGGAGCGGCGACGAGCTGTTTGGGCA
ATCTATGTGTGGTGCAGAAGGACGGATGAGCTAGTGGATGGACCTAATGCTTCATACATTACACCCA
AAGCTCTTGACAGATGGGAGAAAAGACTGACAGATCTCTTCGAAGGCCGGCCTTATGATATGTATGA
TGCTGCTCTATCTGATACAGTCGCCAAGTACCCTGTTGACATTCAGCCCTTCAGAGACATGGTAGAA
GGAATGAGATTAGACTTGAGAAAATCAAGATACCAGAACTTTGATGAACTTTACCTTTACTGCTACT
ATGTTGCTGGAACTGTTGGATTGATGAGTGTTCCGGTAATGGGGATATCCCGGGAATCAAAGGCCTC
AACAGAAAGTGTTTACAATGCTGCATTGGCCCTTGGAATTGCTAATCAGCTCACTAACATTCTCAGA
GATGTTGGAGAAGATGCTAGGAGAGGAAGGATATATCTCCCACAAGACGAGCTTGCCCAAGCCGGCC
TATCAGACGATGACATCTTTCGCGGGAAGGTGACTGACAAGTGGCAAAGTTTCATGAAGGGACAAAT
AAAGAGAGCTAGGATGTTCTTTGATGAGGCTGAGAAGGGTGTCTCAGAGCTCAACTCAGCTAGTAGA
TGGCCAGTATGGGCATCTTTGTTGCTGTACAGGCAGATTCTAGATGCAATTGAAGCAAATGGTTATG
ACAATTTCACAAAAAGGGCATATGTGGGAAAAGCAAAGAAGTTAGCATCATTGCCTGTGGCCTATGG
AAGGGCCATTTTAGGACCCTCTAATTTAACTAAGCAGTTGGTGCCTAGATGA

2/ *Malus domestica* cultivar Granny Smith phytoene desaturase (PDS) protein mRNA, complete cds
GenBank: KU508828.1
CDS: 121..1845

ATGGCGCAGTGGGCTTGTGTCTCCGCTGCTAACTTGAGCTGCCAAGCTACCATCGTAAACACTCAAA
AGCAACGAAACAGTCCCGGATGCGATGCCCTTTCTTTCAAAGGCAGTGAATTTATGGCTCAGAGCTG
TAGATTTTCAAGCCCACAAGCTGTTTATAGAAGGCCCAGGAATGGTGTTTGCCCCTTGAAGGTGGTT
TGCGTTGATTATCCAAGACCAGACCTTGACAGTACTGCTAATTTCTTAGAAGCTGCGTACTTCTCTT
CCACTTTCCGAGCCTCTCCTCGTCCAACCAAGCCGTTAAAAGTTGTGATTGCTGGTGCAGGTTTGGC
TGGTCTGGCAACTGCAAAATATTTGGCGGATGCGGGTCATCAACCTATACTACTAGAAGCGAGAGAT
GTTTTAGGCGGAAAGGTGGCAGCATGGAAAGATAGTGATGGGGACTGGTATGAAACAGGCCTGCATA
TATTCTTTGGGGCATATCCAAATATTCAGAATCTGTTTGGAGAGCTTGGTATTAATGATCGGTTGCA
GTGGAAGGAACATTCTATGATATTTGCAATGCCAAACAAGCCAGGGGAGTTCAGTCGGTTTGATTTC
CTGGAAGTTCTGCCAGCACCCATAAATGGAATATGGGCCATATTGAAGAACAATGAGATGCTGACTT
GTCCAGAGAAAATCAAGTTTGCAATTGGACTACTGCCAGCAATCCTTGGTGGGCAGGCTTATGTTGA
AGCCCAAGATGGCTTGAGCGTAAAAGACTGGATGAGGAAACAGGGCATACCTGATCGAGTAACTACA
GAGGTGTTTATAGCCATGTCAAAGGCCCTTAACTTTATTAACCCTGATGAACTTTCAATGCAGTGCA
TATTGATTGCTTTGAACCGGTTCCTCCAGGAGAAACACGGTTCCAAGATGGCTTTCTTGGATGGTAG
TCCCCCCGAGAGACTCTGTGCTCCAATTGTTGATCATATCCAGTCATTGGGCGGTGAAGTCCGAACT
AATTCCCGAATACAGAAAATTGATCTAAATAACGATGGAACTGTGAAGAGTTTTGTACTAAATAATG
GGAGCGTGATTGAAGCAGATGCGTATGTGTTCGCCACTCCAGTTGATATCCTAAAGCTTCTATTGCC
TGAAAACTGGAAAGAGATGCCATATTTCAAGAAATTGGAGAAATTAGTTGGAGTTCCAGTTATCAAT
GTTCACATATGGTTCGACAGAAAGCTGAAGAACACATATGATCACCTACTTTTTAGCAGAAGTCCTC
TTTTAAGTGTGTATGCTGACATGTCCGTAACATGTAAGGAATATTACAATCCAAACCAATCTATGCT
GGAGTTGGTTTTTGCACCGGCAGAAGAATGGATTTCATGTAGTGATTCTGAAATTATTGATGCTACA

CTCAAAGAACTTGCAAAACTCTTTCCTGACGAAATAGCTGCAGATCAGAGCAAAGCAAAGATTTTGA
AGTACCATGTTGTGAAAACACCAAGGTCTGTTTACAAGACTGTACCAGGTTGTGAACCTTGCCGTCC
CTTGCAAAGATCTCCCCTAGAGGGTTTCTATTTAGCTGGTGATTACACAAAACAAAAGTATTTAGCC
TCAATGGAAGGAGCAGTTCTATCAGGGAAACTTTGTGCTCAGGCGATTGTACAGGATTATGAATTGC
TTGCTGCCCGGGGAATAAAAACAACGTTGGCTGAGGCAGCCGCTCGATGA

3/ *Malus x domestica* zeta-carotene desaturase ZDS mRNA, complete cds
GenBank: AF429983.1
CDS: 124..1830

ATGGCTTCTTGGGCTCTTTTACCGGCAGCTCCAGTAACTGGTCGCTGTCTGGTGATTCCTGCCAGGA
CAAAGAGGTCCTCCTCCTTCTGGGTTCGCTCTTCTTTGGACACTAATGTTTCTGACATGAGGGTAAA
CGGGTTGTTTCCACCTGAACCAGAGTTCTACCGCGGTCCGAAGCTGAAAGTGGCCATTATCGGAGCT
GGACTCGCGGGCATGTCAACCGCCGTTGAGCTTTTGGATCAAGGCCATGAGGTGGATATTTATGAAT
CGAGGCCTTTCATTGGCGGAAAAGTGGGCTCTTTTGTTGATAAAAAAGGAAACCACATTGAAATGGG
ACTCCATGTTTTCTTTGGTTGCTACAGTAATCTTTTCCGATTAATGAAAAAGGTGGGTGCAGACGAA
AATCTTCTTGTCAAGGATCATACTCACACTTTTGTAAACAAAGGGGGTAACATTGGTGAACTTGATT
TTCGGTTCCCAATTGGAGCACCAATACATGGGATTCTTGCATTTTTGTCTACAAATCAGATTAAGAC
TTACGATAAAGCAAGAAATGCAGTGGCTCTTGCCTTAAGTCCGGTTGTAAAGGCTCTTGTTAATCCA
GATGGAGCATTGCAGGACGTACGGAATTTGGATAGTATAAGCTTCTCTGATTGGTTCTTGTCCAAAG
GTGGCACGCGAATGAGCATCCAGCGGATGTGGGATCCTGTTGCATATGCACTTGGGTTTATTGACTG
TGATAATATTAGCGCTCGTTGTATGCTCACTATATTCACATTGTTTGCCACTAAGACCGAGGCTTCC
CTTCTACGCATGCTCAAGGGTTCGCCAGATGTTTACTTAAGTGGCCCCATCAGAGATTATATCATTG
CCAAGGGGGGCAGGTTTCATCTCAGGTGGGGATGTAGAGAAATACTATATGATAAATCTTCTGATGG
CGAAACATATGTTACTGGATTTTCGATGTCTAGGGCTACTAACAAGAAAATTGTGACAGCCGACGCT
TATGTTGCAGCGTGTGATGTGCCTGGAATCAAGAGACTGCTTCCTTCTCAGTGGAGGGAATGGAATT
TTTTCAATAATGTTTATGAGCTAGTTGGAGTCCCTGTTGTCACTGTGCAACTTAGATACGACGGTTG
GGTCACAGAGTTACAAGATCTAGAACGGTCAAGGCAATTGAAGCAAGCTTCGGGATTAGATAATCTC
CTATATACTCCTGATGCAGATTTCTCTTGCTTTGCCGACCTAGCGCTTACTTCTCCTGAAGATTACT
ACATCGAGGGACAAGGTTCACTCCTCCAATGCGTTTTGACACCAGGTGATCCTTACATGCCTTTACC
AAACGAAGAAATTATAGCAAGAGTGACGAAACAGGTTTTGGCTTTATTCCCATCATCCCAAGGTTTA
GAAGTCACTTGGTCATCAGTTGTCAAAATTGGGCAATCTCTCTATCGGGAGGGACCTGGCAAAGATC
CGTTTAGACCTGATCAGAAGACACCTGTGAAGAATTTTTTCCTCGCTGGCTCATACACAAAACAGGA
CTATATCGACAGCATGGAAGGAGCAACTTTGTCTGGCAGGCAAGCCTCTGCATATATATGTGATGCC
GGGGAAGAGTTGGTGGGATTGAGAAAGAAGCTTGCAGCCCAAGATTCTGGGGAATACACAAAAGCTG
TTAATACTACTGATGAGCTGAGTCTTGTCTA

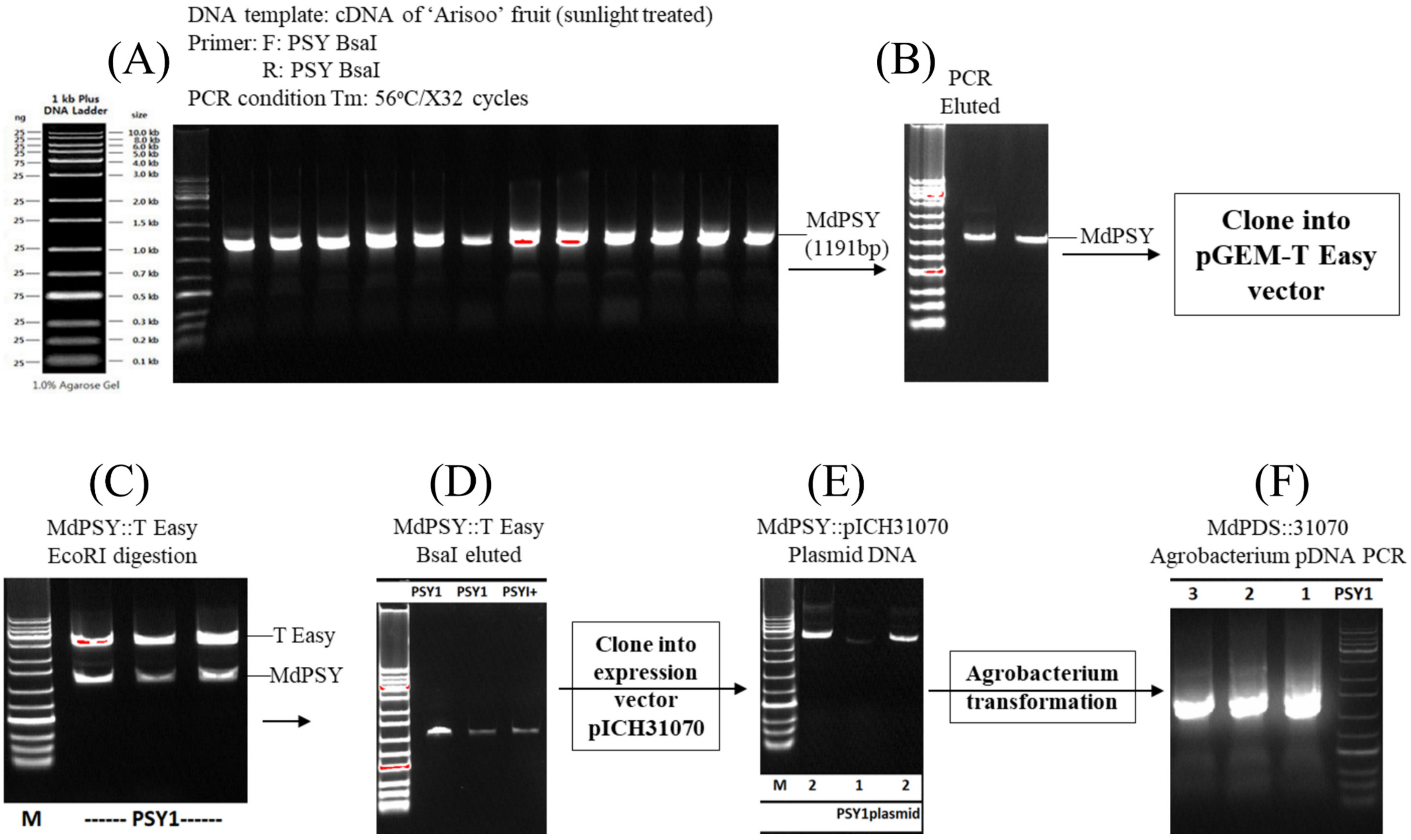

Figure S2. Gene cloning and *Agrobacterium* transformation of ***MdPSY*** gene. (A) *MdPSY* gene synthesis by PCR from the cDNA of peel tissues of ‘Arisoo’ as DNA template using the specific primers apple. (B) *MdPSY* gene were recovered an then cloned into pGEM Teasy vector. (C) Plasmid DNA of MdPSY:: pGEM Teasy were confirmed by EcoRI digestion. (D-E) *MdTFL1::*pGEM Teasy was digested with BsaI and then cloned into expression vector pICH31070 resulted MdPSY::pICH30170. (F) PCR of transformed *A. tumefaciens* EHA105 plasmid DNA.

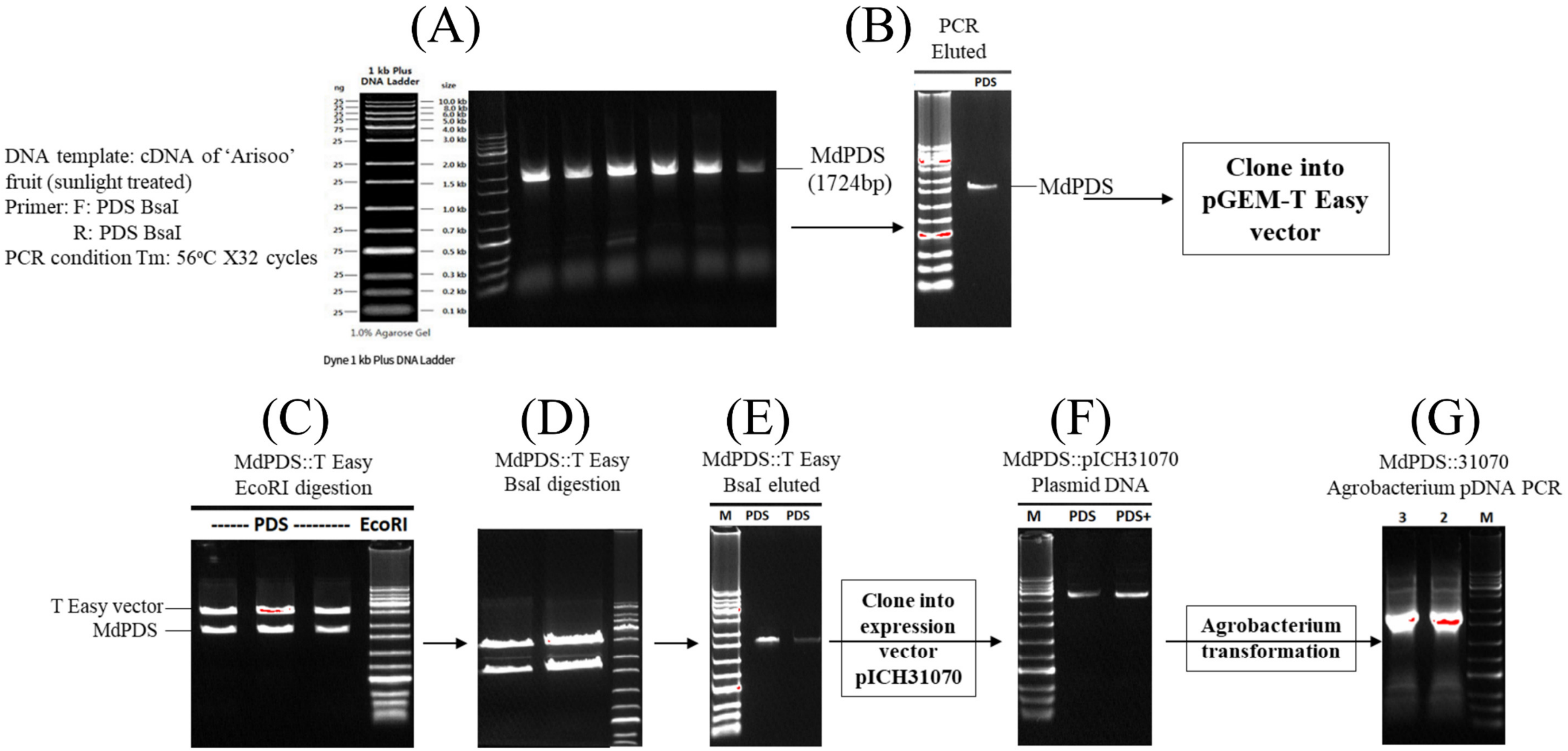

Figure S3. Gene cloning and *Agrobacterium* transformation of ***MdPDS*** gene. (A) *MdPDS* gene synthesis by PCR from the cDNA of peel tissues of 'Arisoo' as DNA template using the specific primers apple. (B) *MdPDS* gene were recovered and then cloned into pGEM Teasy vector. (C) Plasmid DNA of MdPDS::pGEM Teasy were confirmed by EcoRI digestion. (D-F) *MdTFL1::*pGEM Teasy was digested with BsaI, recovered from gel, and then cloned into expression vector pICH31070 resulted MdPDS::pICH30170. (G) PCR of transformed *A. tumefaciens* EHA105 plasmid DNA.

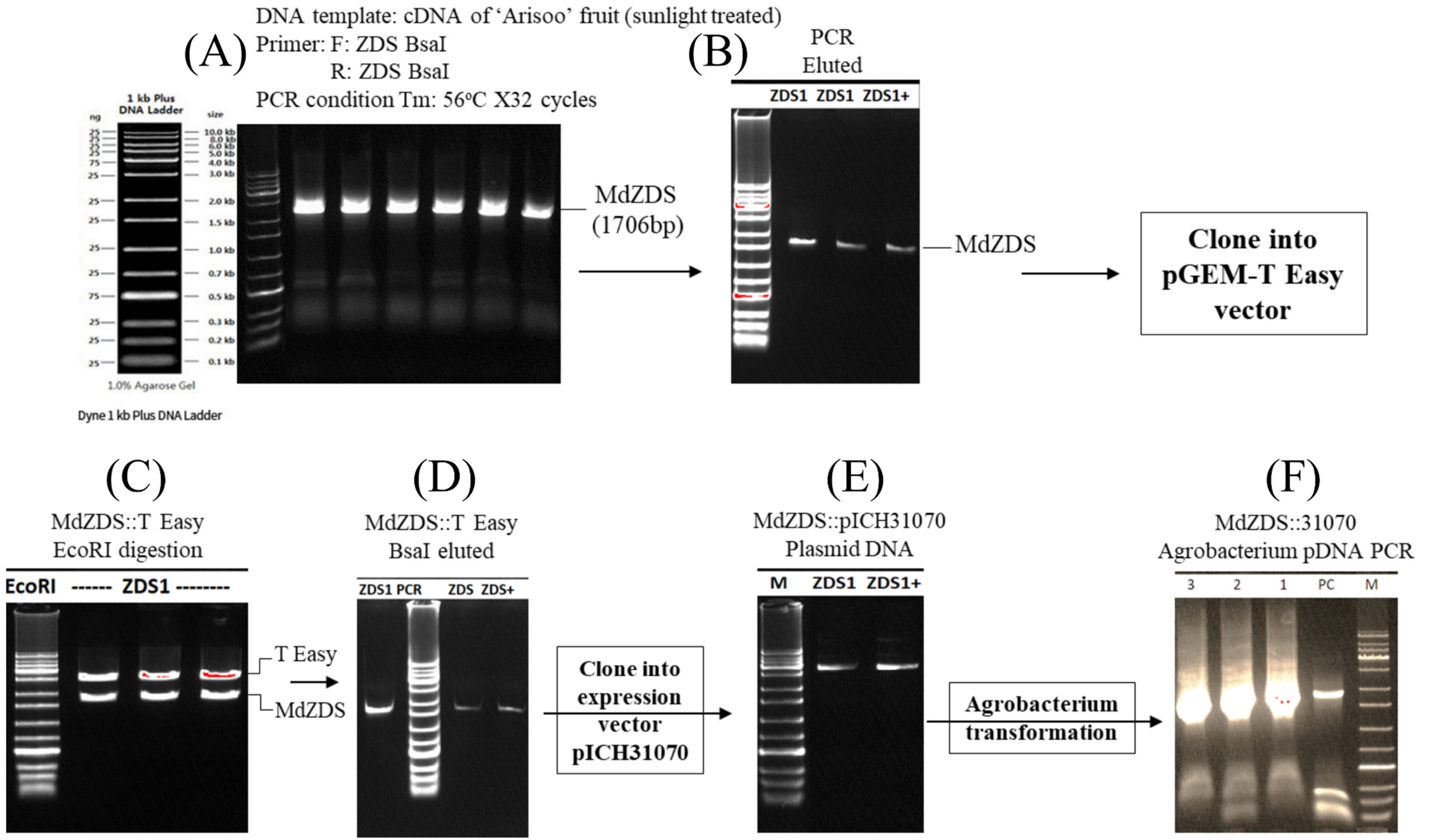

Figure S4. Gene cloning and Agrobacterium transformation of ***MdZDS*** gene. (A) *MdZDS* gene synthesis by PCR from the cDNA of peel tissues of 'Arisoo' as DNA template using the specific primers apple. (B) *MdZDS* gene were recovered and then cloned into pGEM Teasy vector. (C) Plasmid DNA of MdPDS::pGEM Teasy were confirmed by EcoRI digestion. (D) *MdTFL1::*pGEM Teasy was digested with BsaI, recovered from gel, and then (F) cloned into expression vector pICH31070 resulted MdZDS::pICH30170. (G) PCR of transformed *A. tumefaciens* EHA105 plasmid DNA.

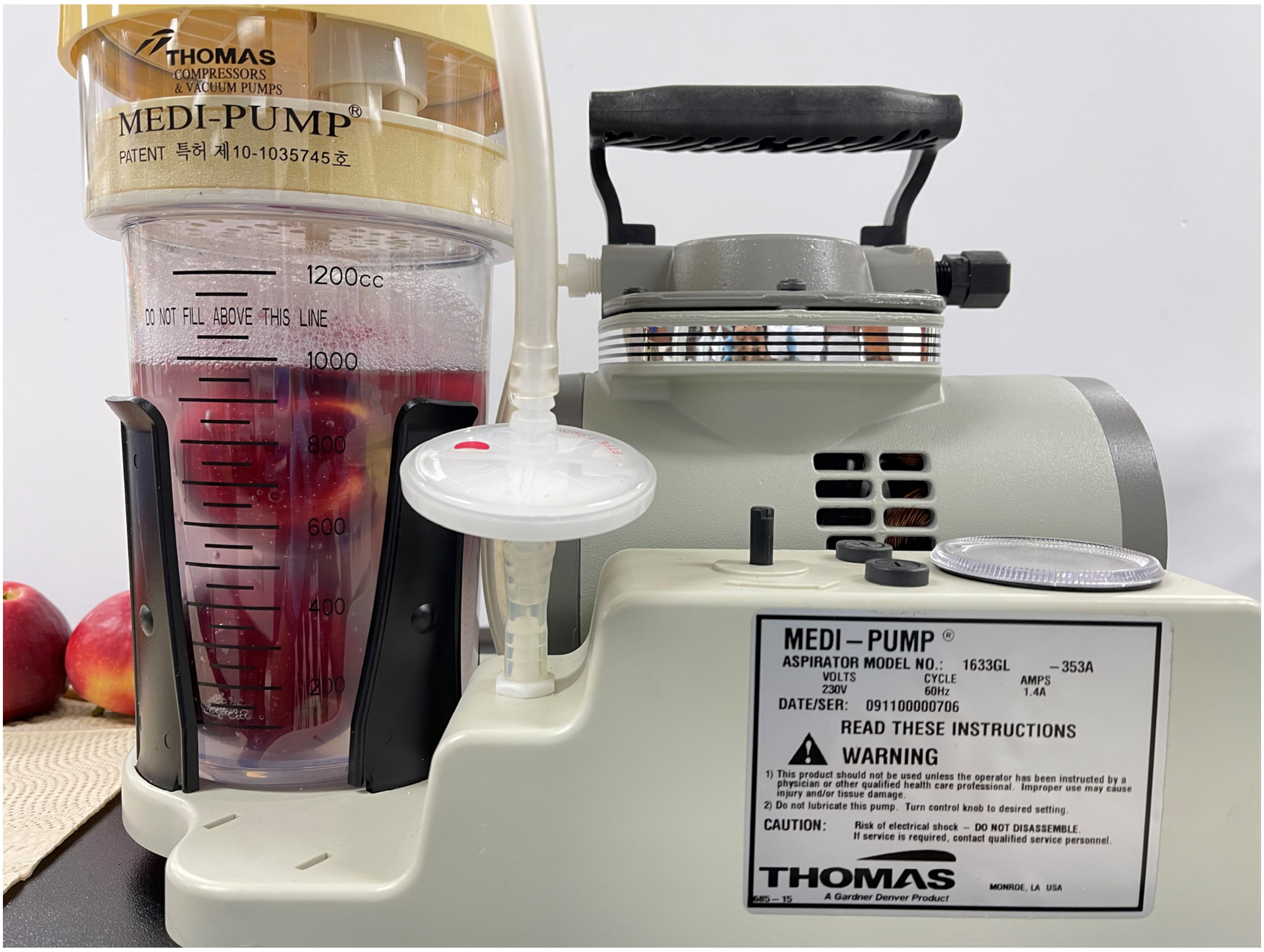

Figure S5. *Agrobacterium* vacuum-infiltration in ‘RubyS’ apple fruit
